# Walking

**Published:** 1874-10

**Authors:** 


### WALKING.

During a warm, sultry day in August, a man walked around Lake Mahopac fourteen and a half times in twenty-four hours, being a distance of eighty miles; during the time he lost fifteen pounds in weight. This loss is, in the shape of perspiration, nearly equal to two gallons. This perspiration consisted of solid particles of the body which had been converted into a liquid form by certain physiological processes; these particles were made up of the waste and useless matters, which, having subserved their purposes, required to be removed, as ashes from a grate; otherwise the wheels of life would have been so clogged up that they could not run, and the machine would have stood still, just as a watch, which for a long time had not been cleaned. A large portion of this waste matter is the result of friction. There are several hundreds of muscles and tendons and pulleys in the human body in unceasing motion, many of them from the cradle to the grave. All other machines wear entirely out during the time, and even then have to be often stopped for repairs; but this wonderful mechanism of the Infinite One makes its own repairs while running, from the material derived from what we eat and drink.

In ordinary life, a man throws off several pounds of waste every day, the greater in amount in proportion to his exercise or working. But the more he throws off, the more he must eat, the more hungry he gets; thus it is that the best way to get a good appetite is to walk or work for it; and if this is done every day, new particles are supplied every day in place of the old, and the body is always new—that is, young, fresh and vigorous; hence Mr. Keys found himself hale and hearty the next day, and was ready for another walk. Thus it is that a daily, cheerful walk would cure half of our maladies, by working off and out of the system the waste and useless matters which cause disease.

